# CDNF Exerts Anxiolytic, Antidepressant-like, and Procognitive Effects and Modulates Serotonin Turnover and Neuroplasticity-Related Genes

**DOI:** 10.3390/ijms251910343

**Published:** 2024-09-26

**Authors:** Anton Tsybko, Dmitry Eremin, Tatiana Ilchibaeva, Nikita Khotskin, Vladimir Naumenko

**Affiliations:** The Federal Research Center, Institute of Cytology and Genetics, Siberian Branch of the Russian Academy of Sciences, Novosibirsk 630090, Russia; eremin@bionet.nsc.ru (D.E.); ilchibaeva@bionet.nsc.ru (T.I.); khotskin@bionet.nsc.ru (N.K.); naumenko2002@bionet.nsc.ru (V.N.)

**Keywords:** CDNF intracerebroventricular injection, mouse, learning, anxiety, behavioral despair, 5-HT, c-Fos, CREB, UPR gene

## Abstract

Cerebral dopamine neurotrophic factor (CDNF) is an unconventional neurotrophic factor because it does not bind to a known specific receptor on the plasma membrane and functions primarily as an unfolded protein response (UPR) regulator in the endoplasmic reticulum. Data on the effects of CDNF on nonmotor behavior and monoamine metabolism are limited. Here, we performed the intracerebroventricular injection of a recombinant CDNF protein at doses of 3, 10, and 30 μg in C57BL/6 mice. No adverse effects of the CDNF injection on feed and water consumption or locomotor activity were observed for 3 days afterwards. Decreases in body weight and sleep duration were transient. CDNF-treated animals demonstrated improved performance on the operant learning task and a substantial decrease in anxiety and behavioral despair. CDNF in all the doses enhanced serotonin (5-HT) turnover in the murine frontal cortex, hippocampus, and midbrain. This alteration was accompanied by changes in the mRNA levels of the 5-HT1A and 5-HT7 receptors and in monoamine oxidase A mRNA and protein levels. We found that CDNF dramatically increased c-Fos mRNA levels in all investigated brain areas but elevated the phosphorylated-c-Fos level only in the midbrain. Similarly, enhanced CREB phosphorylation was found in the midbrain in experimental animals. Additionally, the upregulation of a spliced transcript of XBP1 (UPR regulator) was detected in the midbrain and frontal cortex. Thus, we can hypothesize that exogenous CDNF modulates the UPR pathway and overall neuronal activation and enhances 5-HT turnover, thereby affecting learning and emotion-related behavior.

## 1. Introduction

In recent decades, neurotrophic factors (NTFs) have been considered attractive for the treatment of various neuropathologies. For example, glial-cell-line-derived neurotrophic factor (GDNF) is the most studied NTF in the context of Parkinson’s disease (PD); already, six clinical trials on GDNF in PD patients have been conducted starting from the early 2000s [1]. The well-studied brain-derived neurotrophic factor (BDNF) is recognized as a potential drug for major depression and bipolar disorder [2,3]. BDNF tropomyosin receptor kinase B (TrkB) is a target for antidepressants [4,5,6]. Moreover, antidepressant-like effects are exerted by the administration of BDNF itself into brain ventricles or the parenchyma [7,8,9,10]. A number of studies have indicated that GDNF is also a target for antidepressants [11,12].

Among numerous NTFs and growth factors, cerebral dopamine neurotrophic factor (CDNF) has attracted special attention. CDNF has a paralog—mesencephalic astrocyte-derived neurotrophic factor (MANF)—and together, they form the unique family of proteins with neurotrophic activities [13]. CDNF has several features that distinguish it from conventional NTFs. First of all, CDNF is an endoplasmic reticulum (ER)-resident protein acting as a modulator of the unfolded protein response (UPR) [14]. The three major UPR pathways induced by UPR sensors, called inositol-requiring enzyme 1α (IRE1α), protein kinase R (PKR)-like endoplasmic reticulum kinase (PERK), and activating transcription factor 6 (ATF6), are known to be suppressed by CDNF treatment both in vitro and in vivo [15,16]. Glucose-regulated protein 78 (GRP78, alias: BiP) binds to UPR sensors under homeostatic conditions but dissociates and initiates UPR signaling under ER stress. The dissociation of GRP78 from IRE1α leads to IRE1α dimerization and phosphorylation along with the activation of serine/threonine kinase and related pathways. IRE1α activation enhances its endoribonuclease activity, which triggers the splicing of X-box-binding protein 1 (XBP1) mRNAs. A protein synthesized on spliced *XBP1* (*sXBP1*) mRNA acts as a transcription factor that induces the expression of UPR target genes. CDNF interacts with ER luminal proteins including GRP78, and in this way, can modulate *XBP1* mRNA splicing [15]. Nonetheless, recent in vivo findings suggest that the role of CDNF in UPR regulation in the brain is less prominent compared to the role of MANF [17]. Unlike other NTFs, CDNF has a C-terminal KTEL sequence that anchors it in the ER lumen [18]. In response to ER stress, CDNF expression and secretion are significantly upregulated. It is important to note that specific plasma membrane receptors of CDNF have not been identified yet. Nevertheless, some studies on cardiomyocytes suggest that the KDEL receptor is the cell surface receptor for extracellular CDNF [19]. In additional, the presence of the saposin-like protein (SAPLIP) lipid-binding domain in CDNF enables direct interaction with lipids for entry into the cell by endocytosis [13].

Besides the modulation of ER stress, CDNF differs from other NTFs by its anti-inflammatory properties [20,21,22,23,24]. Recently, it was demonstrated that CDNF can directly interact with α-synuclein, inhibit its cell entry, and reduce protein aggregation [25]; these properties are not typical for conventional NTFs either. All the above-mentioned features make CDNF a potential therapeutic agent for diseases associated with ER stress and proteinopathies. Indeed, CDNF has manifested neuroprotective and neurorestorative effects in animal models of PD [25,26,27,28,29,30,31,32,33,34], Huntington’s disease [35], amyotrophic lateral sclerosis [16], peripheral nerve damage [20,21,36], and ischemic [37] and hemorrhagic stroke [24]. Recently, a phase I trial of human CDNF in PD patients was completed and revealed the safety and tolerability of intraputaminal CDNF infusions [38].

Despite its name, CDNF action is not restricted to dopaminergic neurons exclusively, and its neuroprotective effects have been registered in motor and sympathetic neurons as well [15,16]. It has been demonstrated that CDNF is essential for the development of enteric neurons in mice [39] and of GABAergic and histaminergic neurons in *Danio rerio* [40]. Based on various experimental data, it is believed that CDNF has no survival-promoting or neurite outgrowth activity in naïve neurons [15,26]. On the other hand, Voutilainen and colleagues have reported that CDNF can activate the phosphatidylinositol 3-kinase/protein kinase B (PI3K/Akt) signaling pathway both in vitro and in vivo, even in intact brains [28]. The latter finding indicates that CDNF’s neurotrophic potential goes beyond the participation in ER homeostasis. Moreover, the introduction of the CDNF protein or a *CDNF* transgene into the hippocampus in APP/PS1 mice (transgenic mice expressing a chimeric mouse/human amyloid precursor protein (AßPP) and a mutant human presenilin 1 (PS1); this is an experimental model of Alzheimer’s disease) improves long-term memory [41]. Lately, it has been demonstrated that CDNF overexpression in the hippocampal neurons of ASC mice—in a model of genetically determined depressive-like behavior—improves spatial learning [42]. Not so long ago, a review by Lõhelaid and co-authors also reported that CDNF alleviates depressive behavior in a monkey model of PD [14]. The above-mentioned data indicate that CDNF has good potential for the alleviation of nonmotor symptoms accompanying various neurodegenerative disorders.

Here, we investigated the effects of the intracerebroventricular (i.c.v.) injection of a recombinant human CDNF in C57BL/6 mice on physiological parameters (sleep and feed and water consumption), different types of behavior (operant learning, anxiety-related, and depressive-like), serotonin (5-HT) turnover, and the expression of key serotonergic genes. Additionally, we assessed the expression and phosphorylation of such neuroplasticity-related proteins as c-Fos and CREB. The mRNA levels of major effectors of the UPR pathway (*Atf6*, *Ire1α*, and *Xbp1*) and *Grp78* were quantified, too.

## 2. Results

### 2.1. CDNF Injection Transiently Affects Body Weight and Sleep in Mice without Influencing Locomotor Activity or Feed and Water Consumption

**Figure 1:**
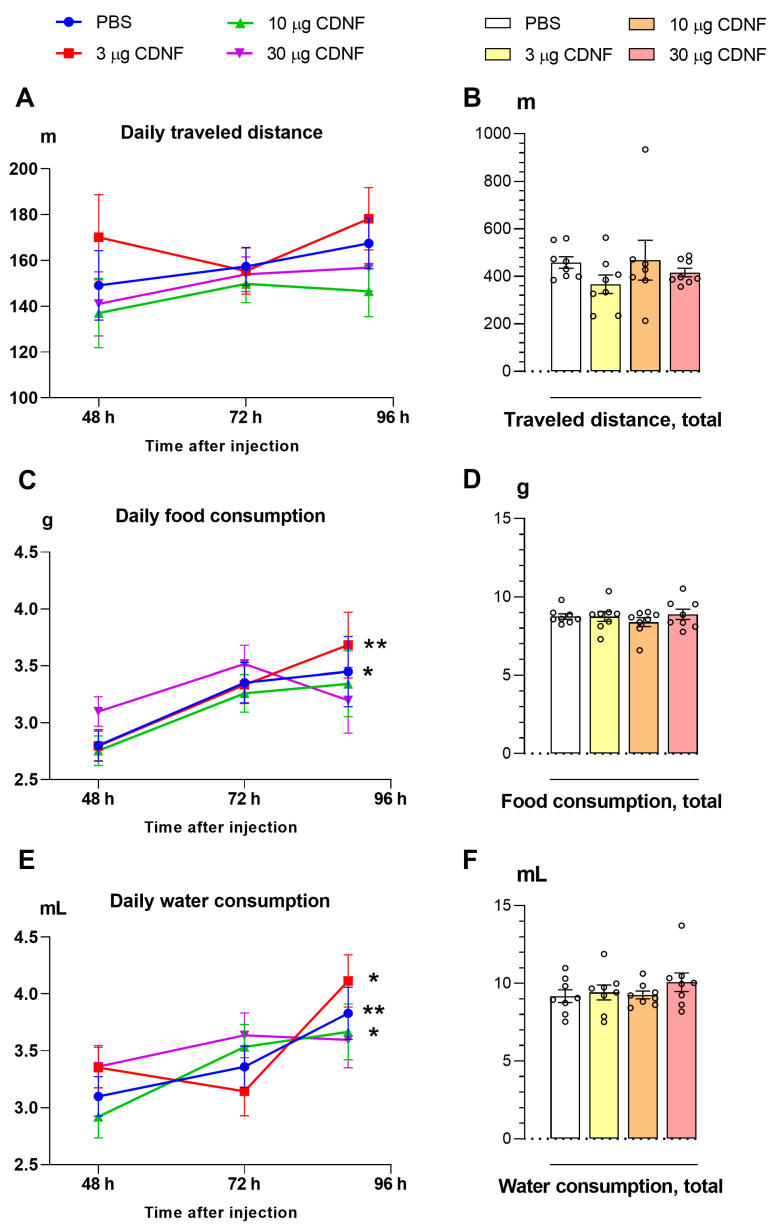
Daily (**A**) and total (**B**) distance traveled were not affected by i.c.v. injection of the CDNF protein. CDNF i.c.v. injection failed to affect either daily (**C**) or total (**D**) feed consumption. * *p* ˂ 0.05 and ** *p* ˂ 0.01 as compared with the first day of the experiment (repeated-measures analysis of variance [ANOVA]). Daily (**E**) and total (**F**) water consumption were not affected by i.c.v. injection of CDNF. * *p* ˂ 0.05 and ** *p* ˂ 0.01 vs. the first day of the experiment (repeated-measures ANOVA). All data are presented as means ± SEMs; *n* ≤ 8.

The single i.c.v. injection of 3, 10, or 30 μg of the CDNF protein failed to affect locomotor activity (Figure 1A,B) or feed (Figure 1C,D) and water consumption (Figure 1E,F) during housing in a home cage for the next 4 days. Only impacts of housing duration on daily feed and water consumption (F_2,54_ = 7.96, *p* < 0.001, and F_2,52_ = 9.60, *p* < 0.001, respectively) were found. Post hoc analysis revealed that at the end of the housing in a home cage, mice in the control group and those treated with 3 μg of CDNF consumed significantly more feed as compared to the first 48 h of this housing (Figure 1C). Similarly, in animals treated with phosphate-buffered saline (PBS) or 3 or 10 μg of CDNF, water intake proved to be elevated at the end of the housing in a home cage (Figure 1E). At the same time, the total water and feed consumption during the entire period of this housing had not been altered (Figure 1D,F). Likely, the observed fluctuations of feed and water intake reflect an ordinary adaptive response to solitary housing.

**Figure 2:**
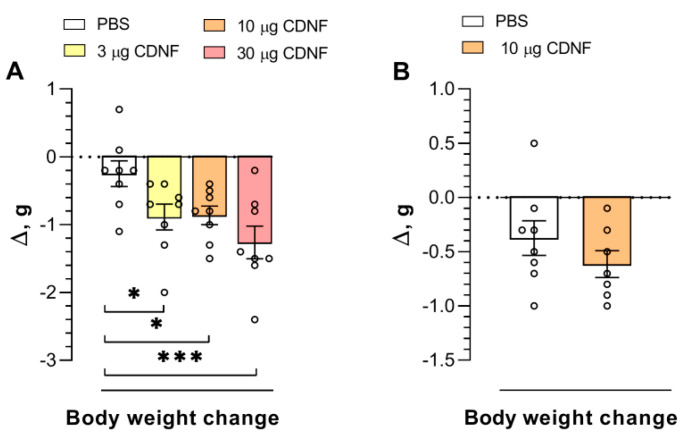
Body weight change (delta, Δ) after PBS or CDNF i.c.v. injection at 4 days (**A**) or 10 days (**B**) post treatment. * *p* ˂ 0.05 and *** *p* ˂ 0.001 vs. the PBS group (one-way ANOVA). The comparison of groups in panel (**B**) was performed with Student’s *t* test. All data are presented as means ± SEMs; *n* ≤ 8.

It is noteworthy that in mice receiving CDNF injections, a decrease in body weight was observed (F_3,28_ = 4.76, *p* = 0.008). The injection of 10 μg of CDNF resulted in a significant (*p* < 0.05) decline in body weight (Figure 2A) measured 4 days after treatment. Nonetheless, the injection of the same dose did not produce such changes 10 days after treatment (Figure 2B). Thus, we can assume that the body weight changes induced by CDNF injection are transient.

**Figure 3:**
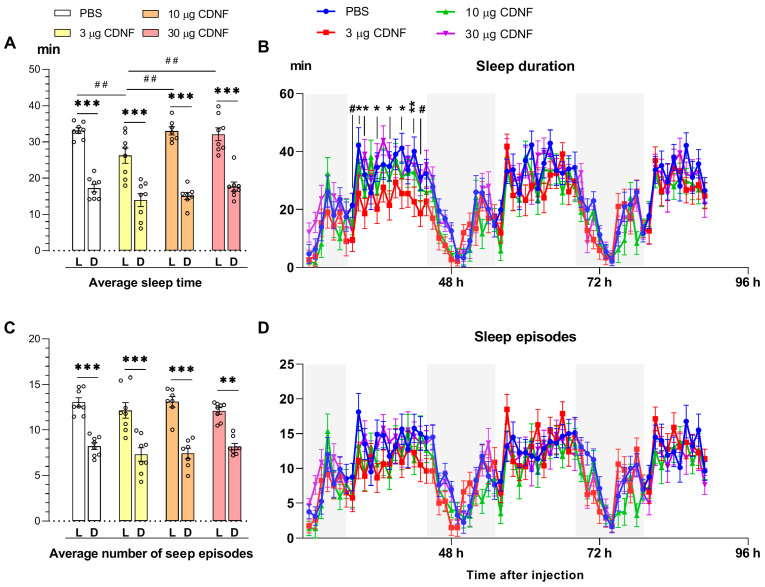
Average sleep duration in the light and dark phases of the day in mice after PBS or CDNF i.c.v. injection (**A**). *** *p* ˂ 0.001 for the dark phase vs. light phase; ^##^ *p* ˂ 0.01 for 3 μg of CDNF vs. other groups (two-way ANOVA). (**B**) Daily dynamics of sleep duration in mice after PBS or CDNF i.c.v. injection throughout the experiment. ^#^ shows marginal significance (*p* = 0.06), * *p* ˂ 0.05, and ** *p* ˂ 0.01 for 3 μg of CDNF vs. the PBS group (repeated-measures ANOVA). (**C**) The average numbers of sleep episodes during the light and dark phases of the day in mice after PBS or CDNF i.c.v. injection. ** *p* ˂ 0.01 and *** *p* ˂ 0.001 for the dark phase vs. light phase (two-way ANOVA). (**D**) Daily dynamics of the numbers of sleep episodes in mice after PBS or CDNF i.c.v. injection throughout the experiment. All data are presented as means ± SEMs; *n* ≤ 8. L: the light phase of the day; D: the dark phase of the day. In panels (**B**,**D**), the dark time of the day is marked with a gray color.

In all animals, a decrease (*p* ˂ 0.001) in the average duration of sleep during the dark time of the day was observed in PBS-treated and CDNF-treated mice on all days of the experiment (Figure 3A), consistently with the known circadian activity of mice [43]. At the same time, a significant influence of CDNF injection was documented (F_3,54_ = 5.53, *p* < 0.01). Mice treated with 3 μg of CDNF demonstrated a significantly shorter (*p* ˂ 0.01) average duration of sleep in the light phase than did mice of other groups (Figure 3A). When we analyzed the daily dynamics of sleep duration, a significant interaction—“i.c.v. injection × time of experiment”—was found (F_192,1792_ = 1.18, *p* = 0.05). Post hoc analysis suggested that during the light phase of the day, in the first 48 h after i.c.v. injection, sleep duration in mice that had received 3 μg of CDNF was diminished, but later, it became normalized (Figure 3B). No effects of CDNF treatment on the number (or daily dynamics) of sleep episodes were detectable. Thus, CDNF, when injected intracerebroventricularly, caused significant but transient sleep disturbances.

### 2.2. CDNF Injection Improves Learning Abilities

**Figure 4:**
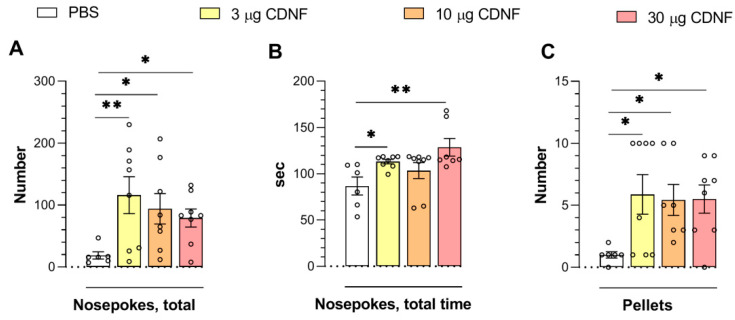
The i.c.v. injection of CDNF improved associative learning in the operant wall, as evidenced by increases in the number of nose pokes (**A**), total time of nose pokes (**B**), and the number of obtained pellets (as reward) (**C**). * *p* ˂ 0.05 and ** *p* ˂ 0.01 vs. the PBS group (one-way ANOVA). All data are presented as means ± SEMs; *n* ≤ 8.

We noticed a significant improvement in learning abilities in animals treated with the CDNF protein. On the third day of the “operant wall” learning task, experimental animals treated with any dose exhibited an increase in the number of nose pokes (F_3,26_ = 3.09, *p* < 0.05; Figure 4A) and in the number of obtained pellets (reward) (F_3,25_ = 3.00, *p* < 0.05; Figure 4C). Furthermore, the total duration of nose pokes was longer (F_3,25_ = 4.55, *p* < 0.05; Figure 4B) in the mice that had received 3 or 30 μg of CDNF.

**Figure 5:**
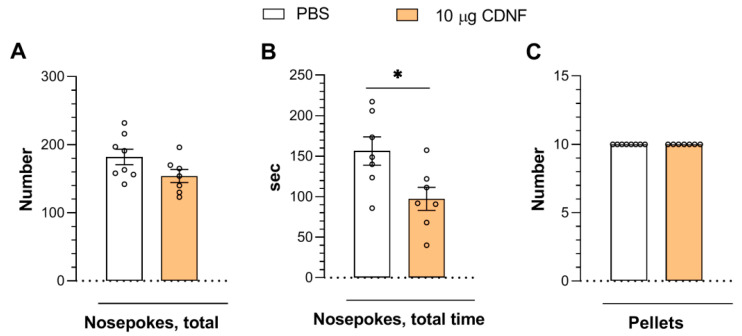
Injection of 10 μg of CDNF did not affect the number of nose pokes (**A**) or the number of pellets obtained (as reward) (**C**) but reduced the total time of nose pokes (**B**) in the operant wall 10 days after treatment. * *p* ˂ 0.05 vs. the PBS group (Student’s *t* test). All data are presented as means ± SEMs; *n* ≤ 8.

In another experiment, 10 days after the injection of 10 μg of CDNF, we detected a slight influence on operant learning. Only the total time of nose pokes was significantly decreased (t = 2.61, df = 12, *p* < 0.05: Figure 5B) in mice injected with CDNF. It is noteworthy that the animals of both groups obtained the maximum number of pellets and exhibited a greater number of nose pokes than in the first experiment (Figure 4). This outcome may be explained by the finding that 4 days after the injection, the animals were still going through the recovery stage, which could have affected their cognitive abilities. Even so, it is clear that mice injected with CDNF demonstrated significantly improved performance. Meanwhile, it should be noted that in the second experiment, the animals that had received 10 μg of CDNF were able to obtain the same number of pellets but in a shorter time (Figure 5B), implying a slight improvement in task performance. Overall, this result indicates that the influence of CDNF is transient and that multiple protein injections or the permanent overexpression via AAV gene therapy constructs are likely required to achieve long-lasting procognitive action.

### 2.3. CDNF Injection Produces Marked Anxiolytic-like and Antidepressant-Like Effects

**Figure 6:**
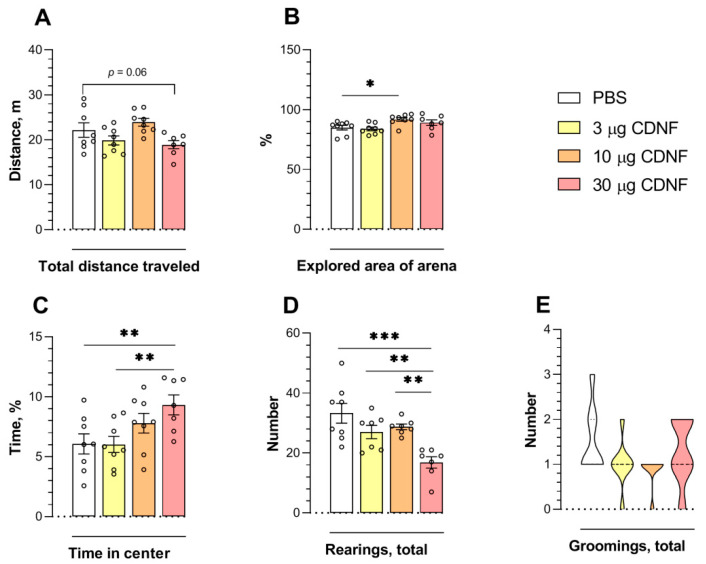
The total distance traveled (**A**), explored area of the arena (**B**), time spent at the center of the arena (**C**), the number of rearings (**D**), and the number of groomings (**E**) in mice after PBS or 3, 10, or 30 μg CDNF i.c.v. injection. * *p* ˂ 0.05, ** *p* ˂ 0.01, and *** *p* ˂ 0.001 (one-way ANOVA). Panels (**A**–**D**) show means ± SEMs, *n* ≤ 8, and panel (**E**) is a violin plot because these data were analyzed by the nonparametric Kruskal–Wallis test.

In the open-field test, we found an effect of the injection on the total distance traveled by the animals (F_3,27_ = 3.77, *p* < 0.05; Figure 6A). Nonetheless, the post hoc analysis revealed only a tendency (*p* = 0.06) toward a decrease in the distance in mice that received 30 μg of CDNF vs. control animals. As demonstrated in the previous experiment (Figure 1A,B), the effect of CDNF on locomotor activity overall was insignificant. The CDNF injection affected the explored area of the arena (F_3,27_ = 4.14, *p* < 0.05). The animals that had received 10 μg of CDNF explored a larger area than control animals did (*p* < 0.05; Figure 6B). We registered the effects of CDNF injection on time spent at the center of the arena (F_3,27_ = 3.83, *p* < 0.05; Figure 6C) and on the number of rearings (F_3,25_ = 8.67, *p* < 0.001; Figure 6D). The CDNF at the 30 μg dose significantly extended the time spent at the center of the arena while diminishing the number of rearings. The longer time spent at the center of the arena indicated an anxiolytic action of the i.c.v. injection of 30 μg of CDNF. Nevertheless, grooming behavior, as another measure of anxiety, was not affected by CDNF injection (Figure 6E). The decrease in the number of rearings may reflect a lesser degree of thigmotaxis rather than weaker exploratory activity because usually, the degree of thigmotaxis negatively correlated with the time spent at the center of the arena.

**Figure 7:**
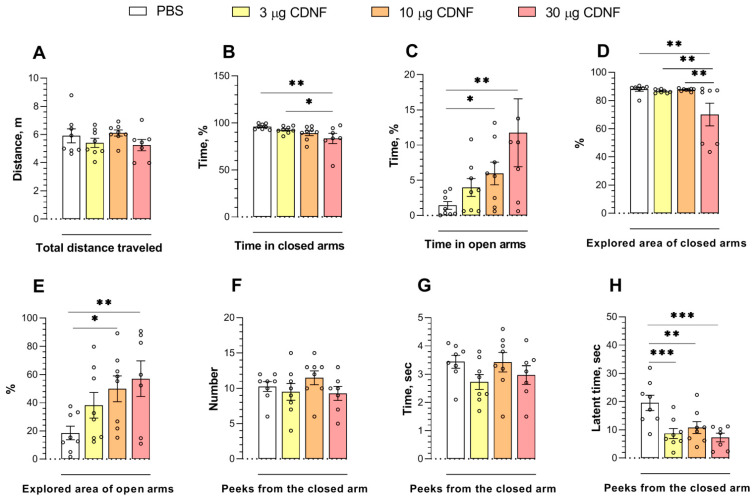
The total distance traveled (**A**), time in closed (**B**) and open (**C**) arms of the maze, explored area of closed (**D**) and open (**E**) arms, and the number (**F**), total duration (**G**), and latency (**H**) of peeks from a closed arm of the maze in mice after PBS or CDNF i.c.v. injection. * *p* ˂ 0.05, ** *p* ˂ 0.01, and *** *p* ˂ 0.001 as compared with the PBS group (one-way ANOVA). All data are presented as means ± SEMs; *n* ≤ 8.

To estimate the anxiolytic-like properties of CDNF protein injection, we performed the elevated plus-maze test. The effects of CDNF were significant for the time spent in the closed (F_3,27_ = 3.32, *p* < 0.05) and open arms (F_3,27_ = 3.11, *p* < 0.05) of the maze and for the explored area in the closed (F_3,26_ = 4.94, *p* < 0.01) and open arms (F_3,27_ = 3.37, *p* < 0.05). The animals that had received 10 or 30 μg of CDNF spent significantly more time in the open arms of the maze (*p* < 0.05 and *p* < 0.01, respectively; Figure 7C). Likewise, the explored area of the open arms of the maze was increased in animals treated with 10 or 30 μg of CDNF (*p* < 0.05 and *p* < 0.01, respectively; Figure 7E). Moreover, the i.c.v. injection of 30 μg of CDNF significantly diminished the time spent in the closed arms (*p* < 0.01; Figure 7B) and explored area of the closed arms of the maze (*p* < 0.01; Figure 7D). Furthermore, we noticed an impact of CDNF injection (F_3,27_ = 6.85, *p* < 0.01) on exploratory behavior: the latency to the first peek from a closed arm was shorter in all mice treated with CDNF (Figure 7H). At the same time, the locomotor activity of animals was not altered (Figure 7A). Thus, the i.c.v. injection of the CDNF protein had a marked anxiolytic-like effect in mice.

**Figure 8:**
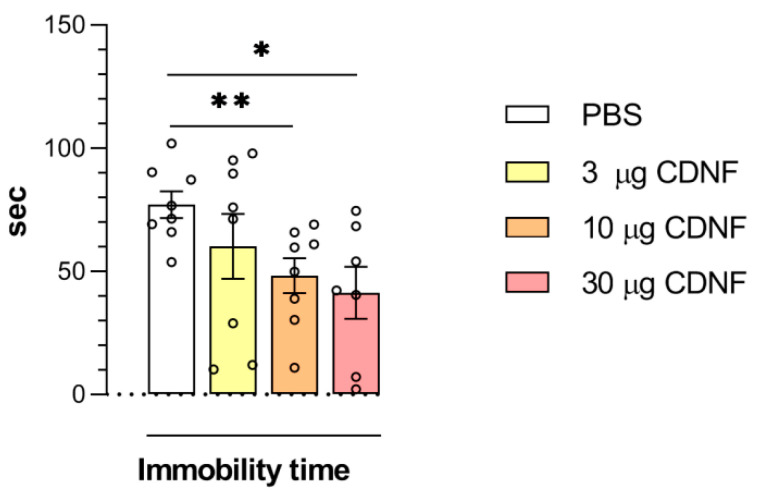
The immobility time was shorter in the forced swim test after i.c.v. injection of the CDNF protein. * *p* ˂ 0.05 and ** *p* ˂ 0.01 vs. the PBS group (unpaired *t* test). All data are presented as means ± SEMs; *n* ≤ 8.

The effect of i.c.v. CDNF injection on depressive-like behavior was analyzed by the forced swim test. Although the main effect of CDNF injection on immobility time in the forced swim test manifested only marginal significance (F_3,27_ = 2.69, *p* = 0.06) according to a one-way ANOVA, the unpaired *t* test revealed a significant decrease in the immobility time in mice treated with 10 or 30 μg of CDNF (*t* = 3.22, df = 14, *p* < 0.01 and *t* = 3.12, df = 13, *p* < 0.01 for 10 and 30 μg, respectively; Figure 8). Consequently, the i.c.v. injection of the CDNF protein into mice produced an antidepressant-like effect that appeared to be dose-dependent.

### 2.4. CDNF Injection Results in Enhanced Serotonin Turnover

**Figure 9:**
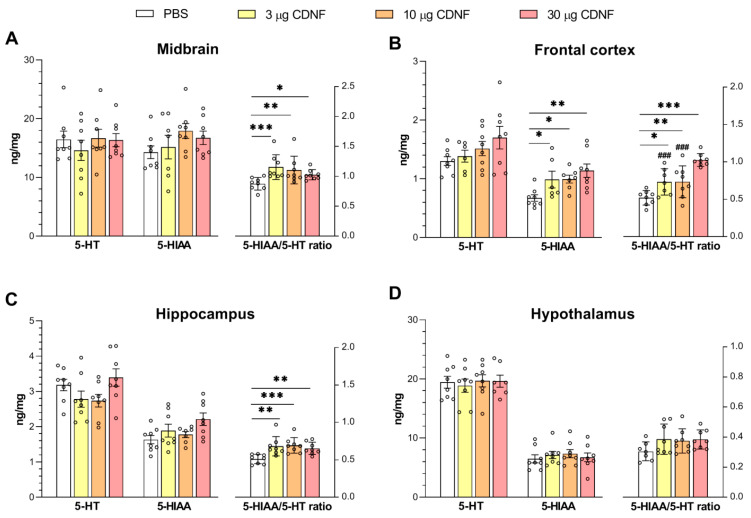
5-HT and 5-HIAA levels and the 5HIAA/5-HT ratio in the midbrain (**A**), frontal cortex (**B**), hippocampus (**C**) and hypothalamus (**D**) of control and CDNF-treated animals. Levels of 5-HT and 5-HIAA are expressed in ng/(mg of total protein). * *p* ˂ 0.05, ** *p* ˂ 0.01, and *** *p* ˂ 0.001 as compared with the PBS group; ### *p* ˂ 0.001 vs. the 30 μg group (one-way ANOVA). All data are presented as means ± SEMs; *n* ≤ 8.

Analysis of 5-HT turnover showed significant changes in 5-HT catabolism, as evidenced by an increase in the 5-hydroxyindoleacetic acid (5-HIAA)/5-HT ratio in the midbrain (F_3,28_ = 4.11, *p* < 0.05; Figure 9A), frontal cortex (F_3,27_ = 3.27, *p* < 0.05; Figure 9B), and hippocampus (F_3,28_ = 5.98, *p* < 0.01; Figure 9C). Additionally, in the frontal cortex, the significant upregulation of 5-HIAA was noted (F_3,25_ = 4.54, *p* < 0.01; Figure 9B). The latter result also supports the notion of enhanced 5-HT catabolism in relevant projection areas after the i.c.v. injection of CDNF.

### 2.5. Expression of Genes Involved in the Reception, Reuptake, and Catabolism of 5-HT Is Affected by CDNF Injection

**Figure 10:**
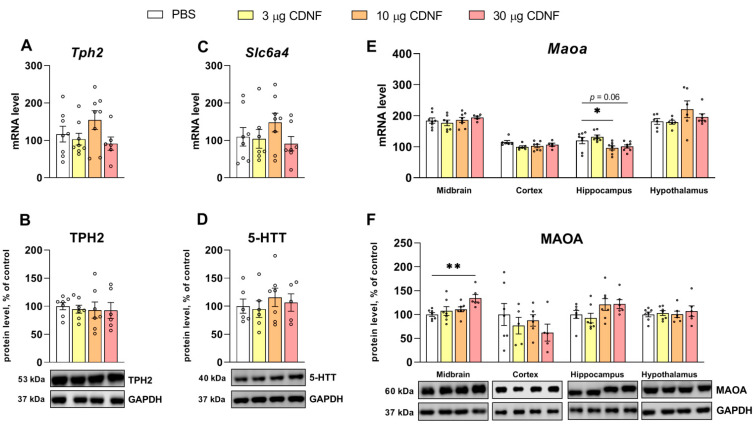
mRNA levels of genes *Tph2* (**A**), *Slc6a4* (**C**), and *Maoa* (**E**), as well as TPH2 (**B**), 5-HTT (**D**), and MAOA (**F**) protein levels after i.c.v. injection of different doses of CDNF or PBS. Each mRNA level is displayed as the number of a gene’s cDNA copies per 100 copies of *Polr2* cDNA. Each protein level is indicated as the ratio of chemiluminescence intensity of a target protein to that of GAPDH. * *p* ˂ 0.05 and ** *p* ˂ 0.01 vs. the PBS group (one-way ANOVA). All data are presented as means ± SEMs; *n* ≤ 8.

We did not find any effects on either the mRNA or protein level of a rate-limiting enzyme of 5-HT synthesis, tryptophan hydroxylase 2 (TPH2) (Figure 10A,B), or a serotonin transporter, 5-HTT (Figure 10C,D), but there was an effect of CDNF protein injection on a major enzyme of 5-HT degradation, that is, the monoamine oxidase A (*Maoa*) mRNA level in the hippocampus (F_3,28_ = 5.45, *p* < 0.01). The post hoc analysis uncovered the downregulation of *Maoa* mRNA in mice treated with 10 or 30 μg of the CDNF protein (Figure 10E). Nevertheless, no corresponding changes were detectable at the protein level. In the meantime, a significant influence of CDNF on the MAOA protein level in the midbrain was observed (F_3,23_ = 4.43, *p* < 0.05): it proved to be elevated in animals treated with 30 μg of CDNF (Figure 10F).

**Figure 11:**
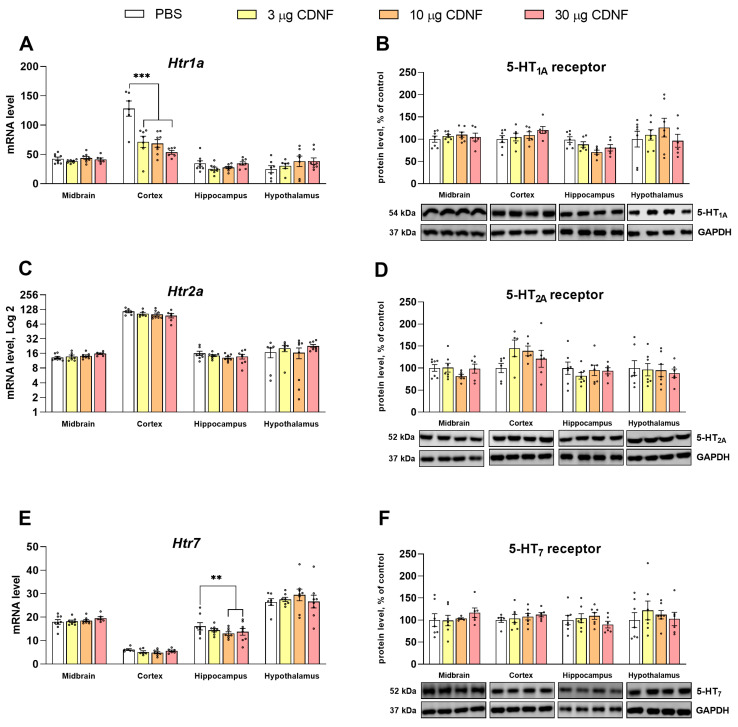
mRNA levels of genes *Htr1a* (**A**), *Htr2a* (**C**), and *Htr7* (**E**), as well as 5-HT1A (**B**), 5-HT2A (**D**), and 5-HT7 (**F**) receptors’ protein levels after i.c.v. injection of CDNF or PBS. Each mRNA level is displayed as the number of a gene’s cDNA copies per 100 copies of *Polr2* cDNA. Each protein level is indicated as the ratio of chemiluminescence intensity of a target protein to that of GAPDH. ** *p* ˂ 0.01 and *** *p* ˂ 0.001 vs. the PBS group (one-way ANOVA). All data are presented as means ± SEMs; *n* ≤ 8.

We noticed significant changes in 5-HT receptors’ mRNA levels in the assayed brain structures. The mRNA level of the 5-HT_1A_ receptor was decreased (F_3,23_ = 13.05, *p* < 0.001) in the cortex in mice treated with any dose of CDNF (Figure 11A). In the hippocampus in mice injected with 10 or 30 μg of the CDNF protein, the mRNA level of the 5-HT_7_ receptor was significantly decreased (F_3,28_ = 5.66, *p* < 0.01 and F_3,27_ = 2.87, *p* = 0.05, respectively; Figure 11E). Nonetheless, there were no changes in the protein levels of any analyzed 5-HT receptors (Figure 11B,D,F).

### 2.6. CDNF Injection Affects the Expression and Phosphorylation of c-Fos and CREB

There is a huge amount of data indicating that c-Fos is a marker of neuronal activation, and this protein is now widely used for tracing neuronal ensembles [44,45]. cAMP response element-binding protein (CREB) plays a central role in molecular cascades underlying synaptic plasticity and long-term memory formation, and its upregulation denotes sustained neuronal activation [46,47,48]. It is known that treatment with the BDNF protein produces an increase in *c-Fos* expression both in vitro [49,50,51,52,53,54] and in vivo [7,55,56]. *c-Fos* upregulation has also been observed after GDNF treatment [56,57,58,59,60,61,62]. The enhancement of CREB phosphorylation after the exposure of neurons to BDNF has been reported [63,64,65,66]. To detect possible similarities with conventional NTFs, we decided to evaluate the expression and phosphorylation of c-Fos and CREB after the injection of CDNF.

**Figure 12:**
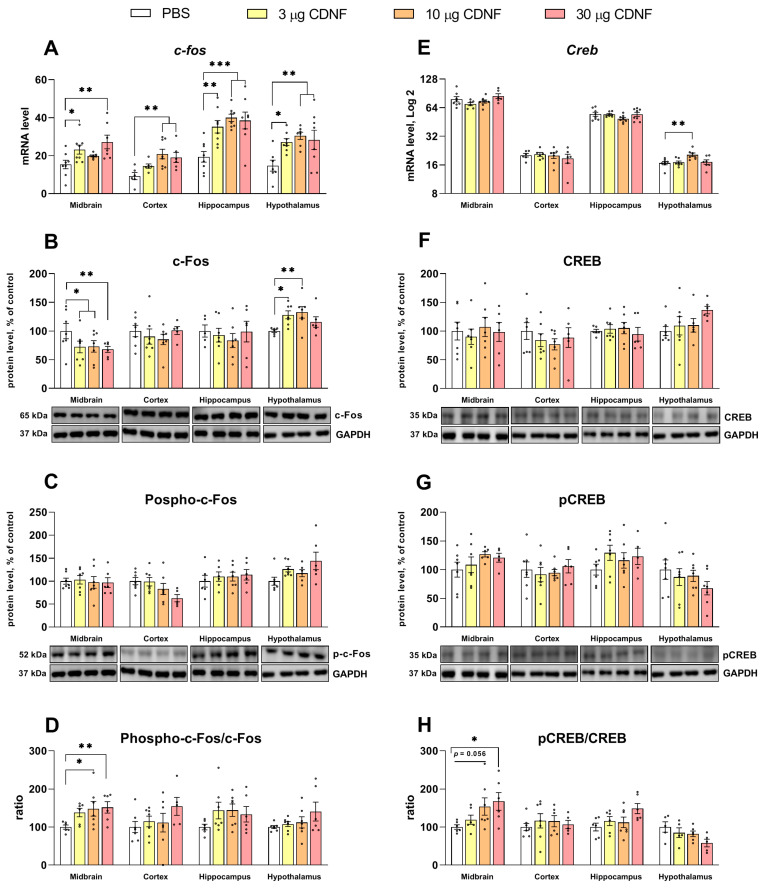
mRNA levels of genes *c-Fos* (**A**) and *Creb* (**E**), as well as c-Fos (**B**), phospho-c-Fos (**C**), Creb (**F**), and phospho-CREB (**G**) protein levels after i.c.v. injection of CDNF or PBS. * *p* ˂ 0.05, ** *p* ˂ 0.01, and *** *p* ˂ 0.001 vs. the PBS group (one-way ANOVA). The phosphorylation of c-Fos and CREB is depicted as the ratio of the nonphosphorylated to phosphorylated protein form (**D**,**H**). * *p* ˂ 0.05 and ** *p* ˂ 0.01 as compared with the PBS group (unpaired t test). The mRNA level is represented by the number of a gene’s cDNA copies per 100 copies of *Polr2* cDNA. Each protein level is indicated as the ratio of chemiluminescence intensity of a target protein to that of GAPDH. All data are presented as means ± SEMs; *n* ≤ 8.

The *c-Fos* mRNA level turned out to be increased in all studied brain structures (F_3,26_ = 4.27, *p* < 0.05; F_3,22_ = 5.5, *p* < 0.01; F_3,27_ = 5.2, *p* < 0.01; and F_3,25_ = 5.75, *p* < 0.01 for the midbrain, frontal cortex, hippocampus, and hypothalamus, respectively; Figure 12A). The mRNA level of CREB was high only in the hypothalamus (F_3,24_ = 4.89, *p* < 0.01; Figure 12E). 

Analysis of c-Fos protein expression showed that in the midbrain, this protein’s level was low (F_3,22_ = 3.88, *p* < 0.05), while in the hypothalamus, upregulation (F_3,22_ = 3.42, *p* < 0.05) was observed (Figure 12B). At the same time, the protein level of phosphorylated c-Fos was unchanged in all the tested brain structures (Figure 12C). The main effect of CDNF injection on the phospho-c-Fos/c-Fos ratio was only marginally significant (F_3,21_ = 2.66, *p* = 0.07) according to a one-way ANOVA. We applied the unpaired *t* test and revealed a significant increase in this ratio in mice treated with 10 or 30 μg of CDNF (*t* = 2.22, df = 11, *p* < 0.05 and *t* = 3.24, df = 10, *p* < 0.01 for 10 and 30 μg, respectively; Figure 12D). Likewise, we failed to detect any differences in either the CREB or phospho-CREB protein level (Figure 12F,G), and the main effect was slightly below significance, but the unpaired *t* test registered a significant increase in this ratio in mice treated with 10 or 30 μg of CDNF (*t* = 2.13, df = 11, *p* = 0.056 and *t* = 2.81, df = 10, *p* < 0.05 for 10 and 30 μg, respectively; Figure 12H).

### 2.7. The Expression of Key Genes Regulating ER Stress Is Affected by Injection of the CDNF Protein

**Figure 13:**
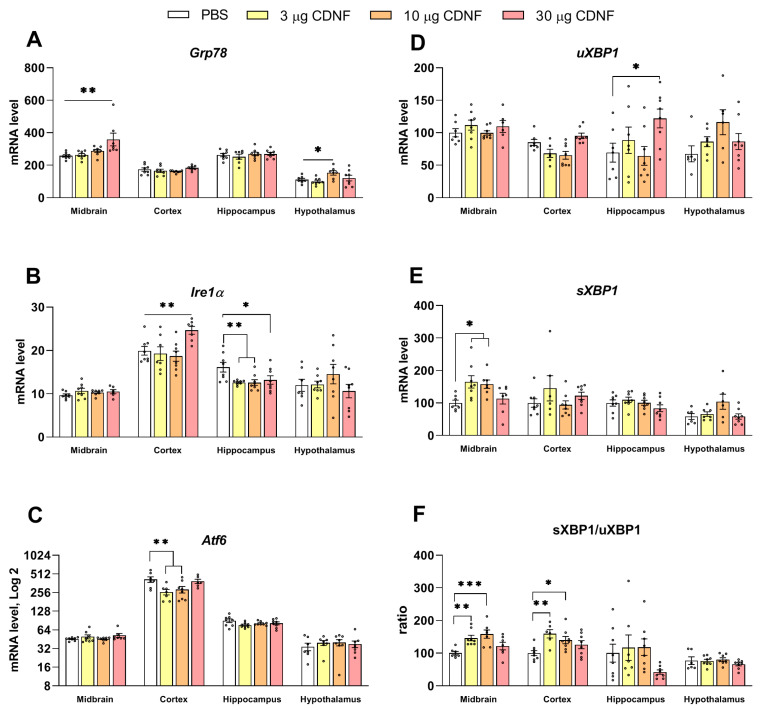
The influence of CDNF injection on both mRNA levels of UPR genes *Grp78* (**A**), *Ire1α* (**B**), *Atf6* (**C**), uXbp1 (**D**), and sXbp1 (**E**) as well as on the sXbp1/uXbp1 ratio (**F**). Each mRNA level (**A**–**C**) is displayed as the number of cDNA copies of a gene per 100 copies of *Polr2* cDNA. In panels (**D**,**E**), expression is depicted as a ratio of sXbp1 and uXbp1 expression levels to the Polr2 expression level. * *p* ˂ 0.05, ** *p* ˂ 0.01 and *** *p* ˂ 0.001 vs. the PBS group (one-way ANOVA, Kruskal–Wallis test for sXbp1 and sXbp1/uXbp1 in the midbrain). All data are presented as means ± SEMs; *n* ≤ 8.

We analyzed the mRNA levels of several key proteins that mediate the UPR and are known to be modulated by CDNF [15]. An impact of CDNF on the *Grp78* mRNA level in the midbrain (F_3,26_ = 5.26, *p* < 0.01) and hypothalamus (F_3,26_ = 3.15, *p* < 0.05) was found (Figure 13A). In the frontal cortex (F_3,26_ = 5.08, *p* < 0.01) and hippocampus (F_3,28_ = 4.27, *p* < 0.05), the effect of CDNF on the *Ire1α* mRNA level was significant (Figure 13B). 

Moreover, we detected a decrease in the *Atf6* mRNA level in the frontal cortex following CDNF injection (F_3,24_ = 6.89, *p* < 0.01; Figure 13C). It is noteworthy that we found both an increase in the level of the sXBP1 transcript in the midbrain (H = 9.44, *p* < 0.05; Figure 13E) as well as an increase in the ratio of sXBP1 mRNA to unspliced XBP1 (uXBP1) mRNA in the midbrain (H = 13.97, *p* < 0.01) and frontal cortex (F_3,26_ = 4.82, *p* < 0.01) in mice treated with CDNF (Figure 13F). It is known that the active form of the XBP1 protein is produced only from a spliced transcript. Therefore, the injection of the CDNF protein could cause XBP1 activation at least in the midbrain and frontal cortex in these experimental animals.

## 3. Discussion

We demonstrated that exogenous CDNF is safe when administered intracerebroventricularly. Previously, the safety and tolerability of a recombinant CDNF protein was documented after intraputaminal infusions both in animal studies and in a clinical trial [38]. A marked weight loss in all animals treated with CDNF was noted in our work. Obviously, this effect was not related to changes in feed and water consumption by the experimental mice. Previously, a decrease in body weight was detected in rats that received acute i.c.v. injections of 10–100 μg of GDNF [67] or continuous injections of 15 μg of BDNF [68]. For BDNF, it is known that its effects on body weight and feed consumption are mediated by the hypothalamic corticotrophin-releasing hormone pathway [68,69]. Thus, we can expect that CDNF may also affect some hypothalamic functions upon i.c.v. injection. It should be pointed out that the effects of CDNF were transient because we could not detect any changes in body weight 10 days after the injection of the moderately effective dose of 10 μg. Nonetheless, it is possible that with repeated CDNF injections, changes in body weight may be more permanent. In our work, the impact of CDNF on sleep duration also, in some way, mimicked the effects of BDNF and GDNF. It has been shown that the i.c.v. injection of BDNF in a dose of 250 ng extends the time of non-rapid-eye-movement (NREM) sleep in rats [70]. Later, it has been demonstrated that the cortical microinjection of BDNF directly modulates sleep homeostasis by strengthening slow-wave activity during NREM sleep [71]. Similarly, the i.c.v. injection of GDNF in a dose of 500 ng increases NREM sleep in rats [72]. Again, our findings indicate a transient effect of CDNF administration on sleep because sleep patterns became normalized 4 days post injection. Of note, after four intraputaminal infusions at monthly intervals, the CDNF protein had no effect on the daytime sleep duration or bouts in MPTP-treated monkeys [73]. Unfortunately, no data were provided by those authors on nighttime sleep quality following the CDNF infusions [73]. Anyway, differences in circadian activity between their animals and ours (monkeys are diurnal animals, and mice are nocturnal), in the targeted brain area, and in doses and regimens of CDNF injection make it hard to compare the findings of the two studies, as do the presence and absence of concomitant MPTP infusion.

We demonstrated the anxiolytic, antidepressant-like, and procognitive effects of a single i.c.v. injection of a recombinant CDNF protein. Earlier, only a study by Kemppainen and coauthors [41] had shown that CDNF protein administration improves learning and memory in mice. This effect was achieved by direct intrahippocampal injection in their study. Furthermore, only in two articles had it been demonstrated that CDNF can improve spatial learning when overexpressed in the hippocampus [41,42]. In contrast to hippocampus-dependent phenomena, such as spatial learning, operant learning involves many cortical and subcortical brain areas [74]. Thus, we demonstrated, for the first time, a positive effect of CDNF protein injection on performance in a complex associative-learning task, implying the involvement of hippocampal, cortical, and striatal neural circuits. In this field, anxiety-like behavior had been previously detected only in CDNF-deficient *D. rerio* [40], but a direct modulatory effect of CDNF on anxiety and behavioral despair is reported for the first time here. Serotonin is the main candidate for the modulation of anxiety, learning, and the psychomotor state because the observed behavioral alterations induced by CDNF injection were accompanied here by significant changes in 5-HT turnover and in the transcription of many genes crucial for the reception and degradation of 5-HT. These effects were very similar to those observed for well-known neurotrophin BDNF after i.c.v. [9,10,75,76,77] or intrahippocampal [8] administration. This was unexpected in the face of the structural and functional differences between BDNF and CDNF. The observed shift in 5-HT catabolism may reflect a possible increase in the synthesis and/or secretion of this neurotransmitter after CDNF injection; these events could have happened in the first hours or days after the treatment.

The significant decline in 5-HT_1A_ gene transcription in the frontal cortex in mice treated with CDNF protein deserves special attention. The prefrontal cortex is rich in 5-HT_1A_ receptors, which are known to inhibit the activity of pyramidal neurons in the cortex [78]. In addition, it is known that 5-HT_1A_ receptors can also be found on GABAergic interneurons in the cortex; these neurons, in turn, can regulate the activity of the serotonergic neurons whose bodies are located in dorsal raphe nuclei in the midbrain [79]. Thus, even postsynaptic 5-HT_1A_ receptors are able to regulate the activity of the 5-HT system through a negative feedback mechanism. In general, the pharmacological activation of 5-HT_1A_ receptors impairs learning and memory, as has been demonstrated in a number of animal studies [80]. Prefrontal 5-HT participates in the regulation of attention [81], and the majority of studies have consistently indicated that an overall reduction in prefrontal 5-HT_1A_ receptor activity accompanied by elevated levels of 5-HT itself is beneficial for attention [78]. It can be hypothesized that a significant decrease in the transcription of the 5-HT_1A_ gene in the frontal cortex in experimental animals will result in at least partially improved associative learning in the operant wall test in CDNF-treated mice. On the contrary, we did not see a dramatic decrease in 5-HT_1A_ receptor protein levels in the frontal cortex in our experimental mice. The 5-HT_1A_ receptor is characterized by strong resistance to internalization [82], slow degradation [83], and fast recovery in the cortical region [84]. By means of Ras-related protein Rab-4A (Rab4) and Ras-related protein Rab-11A (Rab11A) as markers of endosomal intracellular trafficking, it has been shown that the 5-HT_1A_ receptor is recycled back to the plasma membrane in 60 min after incubation with serotonin [85]. These findings may partially explain the discrepancies between 5-HT_1A_ mRNA and protein levels. Not so long ago, it was demonstrated that GRP78 is trafficked to the cell surface via endosomal transport mediated by Rab4 and Rab11 via ER-stress-induced PERK–AKT–mTOR signaling [86]. Whether CDNF can regulate the endosomal trafficking of UPR sensors and G-protein-coupled receptors (such 5-HT_1A_) is still unclear and is a good subject for extensive future research. It is also necessary to mention that some literature data contradict the above-mentioned view on the role of 5-HT_1A_ receptors in memory function. For example, it has been reported that the pharmacological triggering of 5-HT_1A_ receptor in 5-HT-deficient mice reverses memory deficits [87]. Consequently, a change in 5-HT_1A_ receptor expression is not the only mechanism behind the beneficial effects of i.c.v. CDNF injection. A general increase in 5-HT turnover in the brain could reduce anxiety and behavioral despair. This phenomenon could be mediated by other 5-HT receptors, for example 5-HT_2A_ and 5-HT_7_.

MaoA is the main enzyme for 5-HT degradation [88]. Furthermore, MaoA is required for the remodeling of dendrites of pyramidal cells in the basolateral amygdala and orbitofrontal cortex in mice exposed to acute stress [89]. Whether the reduction in *Maoa* mRNA levels in the hippocampus in CDNF-treated animals could be associated with changes in learning and anxiety is controversial. To date, only one study has reported the effect of NTF on *Maoa* transcription in the mouse brain: an increase in the striatal Maoa mRNA level in cataleptic CBA mice following GDNF i.c.v injection [90]. It is likely that changes in hippocampal transcription and midbrain MaoA protein levels are adaptive responses to rapid changes in 5-HT transmission induced by CDNF exposure.

Of interest is the observed substantial increase in the transcription of the immediate–early gene *c-Fos* after CDNF injection. Notably, in our study, the midbrain was the only structure where c-Fos and transcription factor CREB proved to be phosphorylated after CDNF injection. It is possible that this effect may have underlay neuroplasticity changes involving the upregulation of 5-HT synthesis and/or secretion. In addition, 5-HT-induced upregulation in c-Fos expression [91,92,93,94] and CREB phosphorylation [95,96] have been previously reported. Thus, a positive c-Fos/CREB-5-HT-c-Fos/CREB feedback loop cannot be excluded. At the same time, some 5-HT receptors (i.e., 5-HT_1A_ and 5-HT_2A_) are known to affect the synthesis of the c-Fos protein [97,98,99] and phosphorylation of CREB [100]. This may partly explain both the differences between the transcription and phosphorylation of c-Fos and CREB as well as the structure-specific pattern of phosphorylation. Higher CREB phosphorylation after exposure of neurons to BDNF has been reported [63,64,65,66]. On the other hand, BDNF’s effects are transduced through TrkB, which is widely expressed in the area of raphe nuclei and directly in serotonergic neurons [101,102] and induces [103] and regulates [104] their functioning. The influences of BDNF on plasticity-related proteins’ expression and phosphorylation are profound but transient (in the case of single application); for example, *c-Fos* transcription in primary neuronal cultures is upregulated after BDNF application but becomes normalized 3 h later, just like CREB phosphorylation [66]. In contrast, the effects of our single injection of CDNF on c-Fos expression lasted for at least 4 days afterwards in our experiment. Similarly, the phosphorylation of c-Fos and CREB was found to be increased in the midbrain 4 days after the CDNF injection. Despite the apparent similarity of effects between BDNF and CDNF, the mode of CDNF action is strikingly different from that of BDNF. First, CDNF has no specific receptors like TrkB. Second, for CDNF, a major way to modulate neuronal function is intracellular, through ER, e.g., IRE1α signaling [15]. Given that ER stress sensors such as IRE1α affect Ca^2+^ transport and distribution [105], their modulation gives CDNF a powerful tool for the stable upregulation of many genes including *c-Fos* and *Creb*.

We noticed the predominance of sXBP1 mRNA in the midbrain and cortex in CDNF-treated mice. Although changes in the expression levels of the other UPR effectors assayed here were ambiguous, sXBP1 overexpression indicates the specific launch of an important UPR pathway known to be specific for CDNF. Our results are in line with previous findings suggesting that CDNF protects neurons by an intracellular mechanism [15]. XBP1 has been shown to be important for contextual memory formation by modulating long-term potentiation and spine density maintenance, acting as a regulator of memory-related genes including BDNF and Kalirin-7 [106,107]. It is doubtful, however, whether the CDNF-induced increase in sXBP1 is related to c-Fos and CREB upregulation. Previously, we have shown that virally transduced CDNF—while localizing exclusively to the ER and activating the IRE1α–XBP1 pathway—does not enhance c-Fos and CREB expression and phosphorylation [42]. Here, we noted that the *Atf6* mRNA level was decreased in the frontal cortex in CDNF-treated mice. It has been reported earlier that in *Atf6*^−/−^ mice, kainate administration causes the excessive induction of c-Fos, indicating stronger neuronal activation during ATF6 deficiency [108]. *Fos* has been characterized as a constitutive ATF6-responsive gene in human mesenchymal stem cells [109]. Thus, there may be a link between *Atf6* transcription and c-Fos expression. Apparently, there must be an additional pathway transmitting CDNF’s effects. It is possible that KDEL receptors—reported to sense extracellular CDNF in cardiomyocytes [19]—may also contribute to the effects of exogenous CDNF in neuronal cells by triggering the PI3K–Akt pathway.

It has been demonstrated elsewhere that a recombinant CDNF protein is very stable at 37 °C, is widespread in the brain parenchyma, and has a half-life of ~5.5 h [27,110]. For comparison, the BDNF protein has a half-life of approximately an hour [111], and GDNF’s half-life is ~6–8 h [112]. CDNF’s pharmacokinetics may allow it to exert effects even in the absence of specific membrane receptors. At the same time, thanks to receptors, classic NTFs may act at nanomolar concentrations and produce marked alterations in neuronal plasticity and animal behavior that are detectable several weeks after injection [10,77,90]. We observed that 10 days after CDNF injection, performance on the operant wall is substantially blunted. This outcome indicates that the behavioral effects of CDNF are short-lived and most pronounced in the postoperative period; these characteristics may be directly linked with CDNF’s function as an ER stress regulator.

## 4. Materials and Methods

### 4.1. Animals

**Figure 14:**
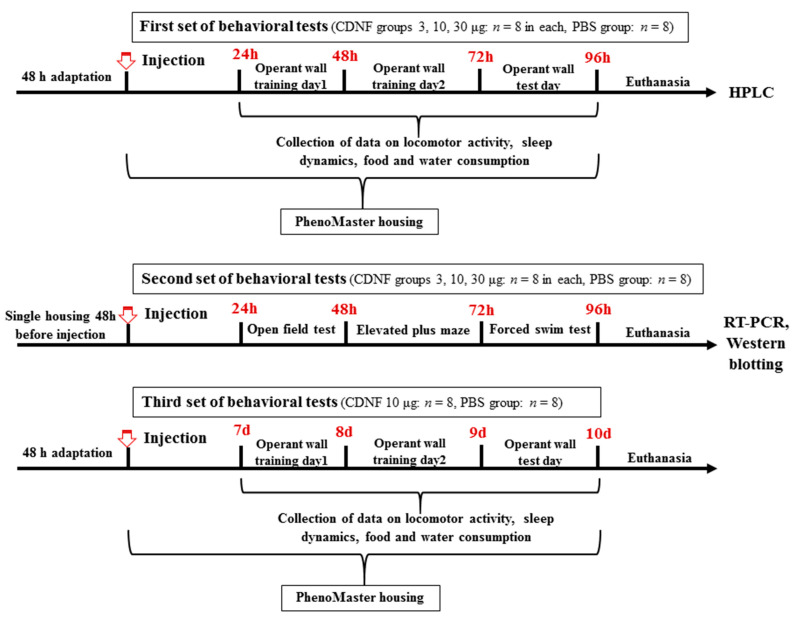
The experimental design.

Specific pathogen-free adult (postnatal day 60, 25 ± 1 g) male mice of the C57BL/6 inbred strain were used. The mice were housed at the Center for Genetic Resources of Laboratory Animals at the Institute of Cytology and Genetics, the Siberian Branch of the Russian Academy of Sciences (ICG SB RAS) (unique identifier RFMEFI62119X0023) under standard laboratory conditions on a 12/12 h light/dark cycle with water and feed available ad libitum. The number of animals in all experimental and control groups in all sets of experiments was 8. In the second set of experiments, 2 days before the i.c.v. injection and behavioral tests, the mice were isolated by placement into individual cages to remove group effects (Figure 14). All surgical procedures were performed under isoflurane anesthesia (3.5% induction for four minutes and 1.5–2.0% maintenance), and every effort was made to minimize the suffering of the animals.

### 4.2. CDNF i.c.v. Injection

The recombinant CDNF protein (Icosagen, Tartu, Estonia) was diluted in sterile PBS and injected in a dose of 3, 10, or 30 μg into the left lateral cerebral ventricle of each mouse (AP: −0.5 mm, ML: −1.0 mm, DV: 2 mm; http://labs.gaidi.ca/mouse-brain-atlas/?ml=-1&ap=-0.5&dv=2, accessed on 1 September 2021). Before this procedure, the animals were anesthetized for 20–30 s with isoflurane. Sterile PBS was injected as a control. The volume of the intracerebroventricularly administered solutions was 3 μL. Behavioral testing was started 1 day after the CDNF injection.

### 4.3. Tests under Home Cage Conditions

Daily dynamics of locomotor activity (m), sleep (min), and water and feed consumption (g) were investigated in the PhenoMaster system (TSE, Bad Homburg, Hessen, Germany). The animals learned how to use drinking bowls for 2 successive days, then they were isolated in PhenoMaster cages and parameters were registered for 65 h. The first 24 h (1–24 h) was considered an adaptive period and was disregarded. All individual cages in the device were equipped with infrared sensors that traced an animal’s movements. The drinking bowls and feeders were also equipped with sensors, allowing for accurate measurement of water and feed consumption. The data from the sensors were recorded each minute and processed by the software (version 7.2.7) from the manufacturer. Immobility of an animal was assessed in periods of 10 s, and the software considered the animal to be sleeping if it recorded four periods of immobility in a row (equivalent to one episode of sleep). Thus, the PhenoMaster software defined the state of sleep as immobility for 40 s or more.

The operant wall was used to estimate the impact of CDNF injection on associative learning in the experimental animals. The operant wall unit has been described elsewhere [113,114]. The beginning of the test always coincided with the beginning of the active wakefulness period of the mice; the signal was light from a light bulb built into the module. The module was programmed in such a way that on the first day, an animal received the simplest task: to receive a reward in the form of a sweet pellet by poking its nose into a hole marked with a switched-on light. On the second day, the task became more difficult: now, in order to receive a reward, it was necessary to perform nose poking into two holes marked with light. On the third day, the task did not differ from the previous one; however, the holes were no longer marked with light: the animal had to remember the necessary sequence of actions on its own. Each day, the test duration was no longer than 120 min. The test ended when the animal obtained all pellets (10 total) or automatically after the specified time interval. Recording of parameters began after the first poke into a hole.

### 4.4. Assessment of Exploratory Activity, Anxiety, and Behavioral Despair

To remove a group effect, the animals were placed into individual cages 2 days prior to the CDNF injection and behavioral tests.

For assessment of exploratory activity, *the open field test* was carried out. A circular arena (40 cm in diameter) bordered by a white plastic wall and illuminated through a mat and semitransparent floor was used. A mouse was placed near the wall and tested for 5 min. The total distance traveled, explored area of the arena, and time spent in the center of the arena were measured automatically by means of the EthoStudio software (version 2.0) [115]. Vertical activity of the animals (the number of rearings) and the number of grooming episodes were determined without the software.

The *elevated plus maze* test was used for anxiety evaluation. The unit consisted of arms that intersected at right angles, two of which were open and two of which were closed. The arms were 30 cm long, 6 cm wide, and 60 cm above the floor. The closed arms had walls made of opaque plastic that were 20 cm high (safety arms). An animal was placed at the intersection of the four arms of the maze facing toward the closed arm. In the next 5 min using the original EthoStudio software, the following parameters were recorded: time spent in open and closed arms and investigated areas of the arms. Peeks from a closed arm were counted manually.

The *forced swim test (FST)* is widely used for assessment of depressive-like behavior in mice and for testing of antidepressant compounds [116,117]. Each mouse was placed in a clear plastic box (15 × 25 cm) filled with water at 25 °C. After 2 min of adaptation, total immobility time was recorded in the EthoStudio software for 4 min [118]. It is well known that mice of the C57BL/6 strain do not exhibit any signs of genetically determined depressive-like behavior. For this reason, we avoid the term depression and instead regard the immobility observed in the forced swim test as a measure of behavioral despair [119].

### 4.5. Excision of Brain Structures

Mice were decapitated 24 h after behavioral testing. To reduce suffering, the mice were anesthetized with carbon dioxide immediately prior to decapitation. Brains were excised on the same day (12:00–14:00 p.m.) and kept on ice; the entire frontal cortex, hippocampus, and midbrain were dissected according to coordinates from an online mouse brain atlas (https://scalablebrainatlas.incf.org/mouse/ABA_v3, accessed on 25 October 2021), frozen in liquid nitrogen, and stored at −80 °C until the RNA or protein isolation procedure. To analyze expression of the genes of interest, we employed brain samples from animals at the second stage of the experiment (Figure 14) who underwent behavioral testing. Brain samples for high-performance liquid chromatography (HPLC) were taken at the first stage of the experiment.

Total RNA was isolated using the ExtractRNA Kit (Evrogen, Moscow, Russia) and treated with DNase without RNase activity (RNase free DNase, Promega, Madison, WI, USA, 1000 o.u./mL). The resulting concentration was determined on an Eppendorf Nanodrop 2000C spectrophotometer (Thermo Fisher Scientific, Waltham, MA, USA). RNA was diluted with sterile water to a concentration of 0.125 μg/μL and stored at −80 °C.

### 4.6. Reverse Transcription

One microgram of total RNA was utilized for cDNA synthesis via mixing with random hexanucleotides (final primer concentration was 5 μM) and 2.25 μmol of sterile KCl. Denaturation at 94 °C for 5 min and annealing at 41 °C for 15 min were performed on a BIS cycler (Koltsovo, Novosibirsk region, Russia). After that, 15 μL of the mixture containing M-MLV reverse transcriptase (200 units) and Tris-HCl (pH 8.3, 0.225 μmol), a mixture of dNTPs (0.015 μmol each), DTT (0.225 μmol), and MnCl_2_ (0.03 μmol) were combined directly on ice. The reaction solution was incubated at 41 °C for 60 min. The synthesized cDNA was stored at −20 °C.

### 4.7. Quantitative Real-Time PCR

PCR was performed using a LightCycler 480 System amplifier (Roche, Rotkreuz, Switzerland). One microliter of cDNA was mixed with 19 μL of a Master Mix (R-402, Sintol, Moscow, Russia). The primers used to amplify cDNA of the studied genes (Table 1) were designed based on sequences published in the EMBL Nucleotide database and synthesized at the BIOSSET company (Novosibirsk, Russia). Serial dilutions of genomic DNA with concentrations of 0.0625, 0.125, 0.25, 0.5, 1, 2, 4, 8, 16, 32, 64, and 128 ng/μL were amplified simultaneously in separate tubes and served as an external exogenous standard to construct a calibration curve. To control specificity of amplification, melting curve analysis was performed for each run with each pair of primers. The calibration curve was generated automatically by the Light Cycler 480 software (Roche Applied Science, Rotkreuz, Switzerland). Gene expression was evaluated as the number of cDNA copies per 100 copies of *Polr2* cDNA [120,121,122]. For sXbp1 and uXbp1, the calculation was carried out by the ΔΔCt method in the thermal cycler’s software (version 1.5).

### 4.8. Western Blot

To assess total protein levels, tissue samples were homogenized in 300 µL of lysis buffer. The buffer consisted of 300 mM NaCl, 100 mM Tris-HCl pH 8.4, 4 mM EDTA, 0.2% of Triton X-100, 1 mM NaVO_4_, 2 mM PMSF, 1 mM mixture of protease inhibitors (chymostatin, leupeptin, antipain, and pepstatin; Sigma-Aldrich, Darmstadt, Germany), and phosphatase inhibitors (PhosSTOP; Roche, Rotkreuz, Switzerland) at the concentration of one tablet per 10 mL of buffer. The homogenate was incubated on ice for 60 min and centrifuged (12,000× *g*, 15 min). The supernatant protein fraction was transferred to a clean test tube and kept at −80 °C. Total protein was quantified by the BCA method using the Pierce BCA Protein Assay Kit (Thermo Fisher Scientific, Waltham, MA, USA). Samples were diluted to concentration of 1500 μg/mL with 2× Laemmli buffer and stored at −20 °C. Samples were heat-treated for protein denaturation (5 min at 95 °C). Protein extracts (15 μg per lane) were separated by SDS-PAGE in a 10% separating gel. For the electrophoresis, a Hoefer SE 600 (Hoefer Inc., Bridgewater, MA, USA) chamber and an EPS 301 power supply (GE Healthcare, Buckinghamshire, UK) were used. The proteins were then transferred to a nitrocellulose membrane (Bio-Rad Laboratories, Hercules, CA, USA) by means of a Trans-Blot Turbo Transfer System (Bio-Rad Laboratories, Hercules, CA, USA) for semidry electroblotting for 2 h at a current of 0.9 A. A mixture of Precision Plus Protein Kaleidoscope Standards (Bio-Rad Laboratories, Hercules, CA, USA) served as a molecular weight marker.

For protein immunodetection, the membrane was blocked for an hour with TBS-T buffer (Tris Buffered Saline, Bio-Rad) supplemented with 0.05% of Tween 20 and 5% of skim milk powder at room temperature and incubated with primary antibodies at 4 °C overnight (Table 2). Next, the membrane was washed five times for 5 min with TBS-T buffer and incubated for an hour with secondary polyclonal antibodies (Table 2) conjugated with horseradish peroxidase at room temperature. The membrane was again washed five times for 5 min with TBS-T buffer.

Bound antibodies were visualized with the Clarity Western ECL Reagent Kit (Bio-Rad) and a C-Digit Blot Scanner (Li-Cor, Lincoln, NE, USA). In each membrane, the constitutively expressed GAPDH protein was quantified as an internal standard for subsequent normalization. Protein expression was expressed in relative units. Intensity of the bands was determined by protein quantification densitometry in Image Studio Lite 5.2 software.

### 4.9. HPLC

An aliquot (50 μL) of an analyzed sample was mixed with 0.6 M HClO_4_ (Sigma-Aldrich, Darmstadt, Germany) containing 200 ng/mL isoproterenol (Sigma-Aldrich, Darmstadt, Germany) as an internal standard. The homogenate was centrifuged at 12,000× *g* for 15 min at 4 °C to precipitate proteins. The pellet was stored at −20 °C until protein quantitation by the Bradford method. The supernatants were diluted twofold with ultrapure water and filtered using a centrifuge tube with a 0.22 μm cellulose acetate filter (Spin-X, Corning, Costar, Glendale, AZ, USA). Twenty microliters of the filtered supernatant was injected into the loop of the HPLC system.

5-HT and 5-HIAA were quantified by HPLC using a system containing an electrochemical detector (700 mV, Antec DECADE IITM Electrochemical Detector; DataApex, Prague, Czech Republic); a glassy carbon flow cell (VT-03 cell 3 mm GC sb; Antec); system controller, CBM-20A; solvent delivery unit, LC-20AD; autosampler, SIL-20A; and degasser, DGU-20A5R (Shimadzu Corporation, Columbia, MD, USA). Chromatographic separation was carried out by isocratic elution at a flow rate of 1 mL/min on a Luna C18 column (5 μm particle size, L.I.D. 100.4.6 mm; Phenomenex, Torrance, CA, USA) protected with a C8 security guard cartridge (Phenomenex). The mobile phase was 10% methanol (HPLC grade; Thermo Fisher Scientific, Waltham, MA, USA) in 50 mM phosphate buffer (Sigma-Aldrich, Darmstadt, Germany) containing 300 μg/L octanesulfonic acid sodium salt (Sigma-Aldrich, Darmstadt, Germany) (pH 3.9). The temperature of the column was stabilized at 40 °C. Concentrations of analytes were expressed in ng/(mg protein) (determined by the Bradford method).

### 4.10. Statistical Analysis

All the data were examined for normality of distribution by Kolmogorov–Smirnov and D’Agostino–Pearson tests. Outliers were excluded by the Dixon Q-test. Time course results from behavioral testing in the home cage (consumption of water or feed, distance traveled, and sleep) were processed by ANOVA for repeated measures followed by Fisher’s post hoc test. Results of the operant wall test and body weight measurements for animals from the third set of experiments were processed by Student’s *t* test or the Mann–Whitney *U* test. The results of the molecular assays were subjected to one-way ANOVA followed by Fisher’s post hoc test. The data are shown as means ± SEMs.

## 5. Conclusions

Our results—together with previous findings about the involvement of CDNF in the development of enteric neurons and in control over the functions of midbrain dopaminergic neurons in mice [39] and brain neurotransmitter systems in *D. rerio* [40], as well as in procognitive effects during CDNF overexpression [41,42]—indicate that CDNF has much more in common with conventional NTFs than previously thought. Meanwhile, CDNF’s influence on the serotonin system and animal behavior may be based on overall neuronal activation and could be nonspecific. Thus, despite the apparent similarity to conventional NTFs such as BDNF, CDNF has a unique mechanism of action. Nevertheless, our findings suggest that CDNF is a modulator of 5-HT turnover, learning, and emotion-related behavior, and this property may be beneficial for the treatment of nonmotor symptoms in neurodegenerative diseases.

## Figures and Tables

**Table 1:** The primer sequences, annealing temperatures, and PCR products’ lengths.

Target Gene	Primer Sequences	Annealing Temperature, °C	Amplicon Length, bp
*Polr2*	F 5′-GTTGTCGGGCAGCAGAATGTAG-3′ R 5′-TCAATGAGACCTTCTCGTCCTCC-3′	61	188
*Htr1a*	F 5′-CTGTGACCTGTTTATCGCCCTG-3′ R 5′-GTAGTCTATAGGGTCGGTGATTGC-3′	62	109
*Htr2a*	F 5′-AGAAGCCACCTTGTGTGTGA-3′ R 5′-TTGCTCATTGCTGATGGACT-3′	61	169
*Htr7*	F 5′-GGCTACACGATCTACTCCACCG-3′ R 5′-CGCACACTCTTCCACCTCCTTC-3′	65	198
*Tph2*	F 5′-CATTCCTCGCACAATTCCAGTCG-3′ R 5′-CTTGACATATTCAACTAGACGCTC-3′	61	239
*Slc6a4*	F 5′-CGCTCTACTACCTCATCTCCTCC-3′ R 5′-GTCCTGGGCGAAGTAGTTGG-3′	63	101
*Maoa*	F 5′-AATGAGGATGTTAAATGGGTAGATGTTGGT-3′ R 5′-CTTGACATATTCAACTAGACGCTC-3′	61	138
*c-Fos*	F 5′-AAAGAGAAGGAAAAACTGGAG-3′ R 5′-CGGAAACAAGAAGTCATCAA-3′	58	264
*Creb*	F 5′-GCTGGCTAACAATGGTACGGAT-3′ R 5′-TGGTTGCTGGGCACTAGAAT-3′	64	140
*Atf6*	F 5′-CTCAAACCAATGCCAGTGTCC-3′ R 5′-ATGCTGATAATCGACTGCTGC-3′	59	94
*Grp78*	F 5′-CGCTCTACCATGAAGCCTGT-3′ R 5′-AGCCTCATCGGGGTTTATGC-3′	60	174
*Ire1α*	F 5′-TCTGGGGATGTCCTGTGGAT-3′ R 5′-CTTGGCCTCTGTCTCCTTGG-3′	60	195
*uXbp1*	F 5′-CAGACTACGTGCACCTCTGC-3′ R 5′-CAGGGTCCAACTTGTCCAGAAT-3′	60	139
sXbp1 (cDNA)	F 5′-GCTGAGTCCGCAGCAGGT-3′ R 5′-CAGGGTCCAACTTGTCCAGAAT-3′	60	130

**Table 2:** Characteristics of antibodies used.

Target Protein	Primary Antibody		Secondary Antibody: Dilution, Manufacturer Code
	Antibody Dilution	Manufacturer Code	
5-HT1A	1:1000	Ab 85615 (Abcam, Cambridge, UK)	Anti-rabbit 1:10 000, G-21234 (Invitrogen, Waltham, MA, USA)
5-HT2A	1:500	Ab 66049 (Abcam, UK)	
5-HT7	1:1000	Ab 128892 (Abcam, UK)	
TPH-2	1:1000	Ab 184505 (Abcam, UK)	
5-HTT	1:1000	303614 (USBiological Life Sciences, Salem, MA USA)	
MAOA	1:1000	Ab 126751 (Abcam, UK)	
CREB p-CREB	1:1000 1:1000	Ab3138 (Abcam, UK) Ab 32096 (Abcam, UK)	
c-Fos p-c-Fos	1:500 1:1000	Sc-52 (Santa Cruz Biotechnology, Santa Cruz, CA, USA) D82c12 (Cell Signaling Technology, Danvers, MA, USA)	Anti-rabbit 1:8000, G21234 (Invitrogen, USA)
GAPDH	1:7000	Ab 8245 (Abcam, UK)	Anti-mouse 1:30 000, ab6728 (Abcam, UK)

## Data Availability

Data are available from the corresponding author upon reasonable request.
